# Development of flow cytometry based adherence assay for *Neisseria gonorrhoeae* using 5′-carboxyfluorosceinsuccidyl ester

**DOI:** 10.1186/s12866-019-1438-2

**Published:** 2019-03-25

**Authors:** Sidharath Dev Thakur, Milan Obradovic, Jo-Anne R. Dillon, Siew Hon Ng, Heather L. Wilson

**Affiliations:** 1 Vaccine and Infectious Disease Organization - International Vaccine Centre (VIDO-InterVac), University of Saskatchewan, Saskatoon, Saskatchewan S7N 5E3 Canada; 2 School of Public Health, Vaccinology and Immunotherapeutics Program, University of Saskatchewan, Saskatoon, Saskatchewan Canada; 3 Department of Microbiology and Immunology, College of Medicine, University of Saskatchewan, Saskatoon, Saskatchewan Canada

**Keywords:** Flow cytometry, *Neisseria gonorrhoeae*, Neutralizing antibodies, 5′-carboxyfluoroscein succidyl ester

## Abstract

**Background:**

*Neisseria gonorrhoeae* is an obligate human pathogen and its adherence to host cells is essential for its pathogenesis. Gonococcal adherence assays are based on the enumeration of bacteria attached to human cells on solid media. Because conventional adherence assays are based on bacterial counts, they are often time consuming to perform and prone to observer bias. A flow cytometry based method, using the cell-permeable fluorescent dye 5′-carboxyfluoroscein succidyl ester (CFSE), was developed to dramatically increase the number of adherent *N. gonorrhoeae* quantified per assay while improving repeatability and removing observer bias. Piliated *N. gonorrhoeae* F62 were stained with CFSE then the staining reaction was quenched with foetal bovine serum. Human cervical ME-180 cells were infected with CFSE-stained *N. gonorrhoeae* (multiplicity of the infection 100:1) for 2 h. Infected cells were washed to remove loosely adhered bacteria. Flow cytometry was used to quantify the percentage of ME-180 cells associated with CFSE-stained *N. gonorrhoeae* and a minimum of 30,000 events were recorded. Real time-PCR analysis targeting *opa* gene (encoding *N. gonorrhoeae* opacity associated gonococcal outer membrane protein) was performed on infected ME-180 cells to confirm the flow cytometric adherence assay results. A rabbit was immunized with heat-killed *N. gonorrhoeae*F62 to generate hyperimmune serum. The functional compatibility of the assay was confirmed by studying the effect of *N. gonorrhoeae* F62 antiserum on blocking adherence/invasion of CFSE-stained bacteria to ME-180 cells.

**Results:**

We observed that 20.3% (+/− 1.0) ME-180 cells were associated with CFSE-stained *N. gonorrhoeae*. Heat-inactivated hyperimmune serum, at 1:10 to 1:80 dilutions, significantly inhibited gonococcal adherence by 6 and 3 fold, respectively. Real time-PCR analysis targeting *opa* gene confirmed that hyperimmune serum blocked adherence/invasion of *N. gonorrhoeae* to the ME-180 cells in a dilution-dependent manner.

**Conclusions:**

Flow cytometric analysis was amenable to quick, easy and high-throughput quantification of the association of *N. gonorrhoeae* with ME-180 cells and was functionally confirmed using PCR analysis. These approaches may be adapted for in vitro and in vivo adherence studies related to gonococcal pathogenesis.

**Electronic supplementary material:**

The online version of this article (10.1186/s12866-019-1438-2) contains supplementary material, which is available to authorized users.

## Background

Flow cytometry-based adherence assays have been used to quantify interactions between bacteria and eukaryotic cells and these assays have numerous benefits over conventional assays [1, 2]. In conventional adherence assays, bacteria are allowed to attach to solid-phase immobilized cells, they are washed and enumerated either by viable counts or by counting the bacteria visible under a microscope. These methods tend to be time consuming, are not always highly repeatable, and are prone to observer bias [1]. Flow cytometric adherence assays allow for the fast and highly reproducible quantification of significantly higher numbers of bacteria/eukaryotic cell interactions.

*Neisseria gonorrhoeae* is an obligate human pathogen that causes gonorrhea. *N. gonorrhoeae* primarily infects mucosal membranes which are lined with non-cornified columnar or cuboidal epithelial cells and are found in the urogenital tract, the rectal mucosa, the conjunctiva and pharynx [3]. In men, infection with *N. gonorrhoeae* primarily manifests as urethritis and can occasionally lead to infertility and acute or chronic prostatitis. In women, cervicitis is the most common clinical manifestation of *N. gonorrhoeae* infection, but untreated infection may lead to pelvic inflammatory disease (PID)and further serious complications of reproductive health such as ectopic pregnancy [3, 4]. Disseminated infections can cause skin and/or joint/tendon infection and, rarely, endocarditis or meningitis [5]. Newborns may become infected during delivery and acquire eye infections (*ophthalmia neonatorum*) or disseminated disease [5]. Gonococcal infection is a major public health threat because it facilitates the transmission and acquisition of Human Immunodeficiency Virus [6] and because gonorrhea may become untreatable as *N. gonorrhoeae* has developed resistance against all classes of antimicrobials [7].

Carboxyfluoroscein diacetate succinimidyl ester (CFDA-SE) is a fluorescent membrane permeable ester that will, upon activation by intracellular esterase, covalently link to intracellular proteins through its succinimidyl group [8]. CFSE stains bacteria efficiently without affecting cell adhesion ability, viability or metabolism and it is retained for long periods of time which makes it amenable to tracking association or invasion of eukaryotic cells even as they replicate [9]. Flow cytometric assays using CFSE bacterial staining had been to used measure in vivo and in vitro bacterium and host/eukaryotic cell interactions [1, 9, 10]. Recently, our laboratory demonstrated that this method is an effective high throughput tool to measure bacterial infection of eukaryotic cells [10]. Here in we developed a carboxyfluoroscein (CFSE) staining-based flow cytometric assay to quantify gonococcal adherence to human cervical ME-180 cells, a cell line used extensively to study *N. gonorrhoeae* and host cell interactions [10]. Flow cytometry adherence results were confirmed by RT-PCR analysis. Anti-*N. gonorrhoeae* hyperimmune serum antibodies inhibited gonococcal adherence to ME-180 cells and were used to determine the functional compatibility of the assay.

## Results

### CFSE staining of *N. gonorrhoeae* and bacterial viability

**Fig. 1:**
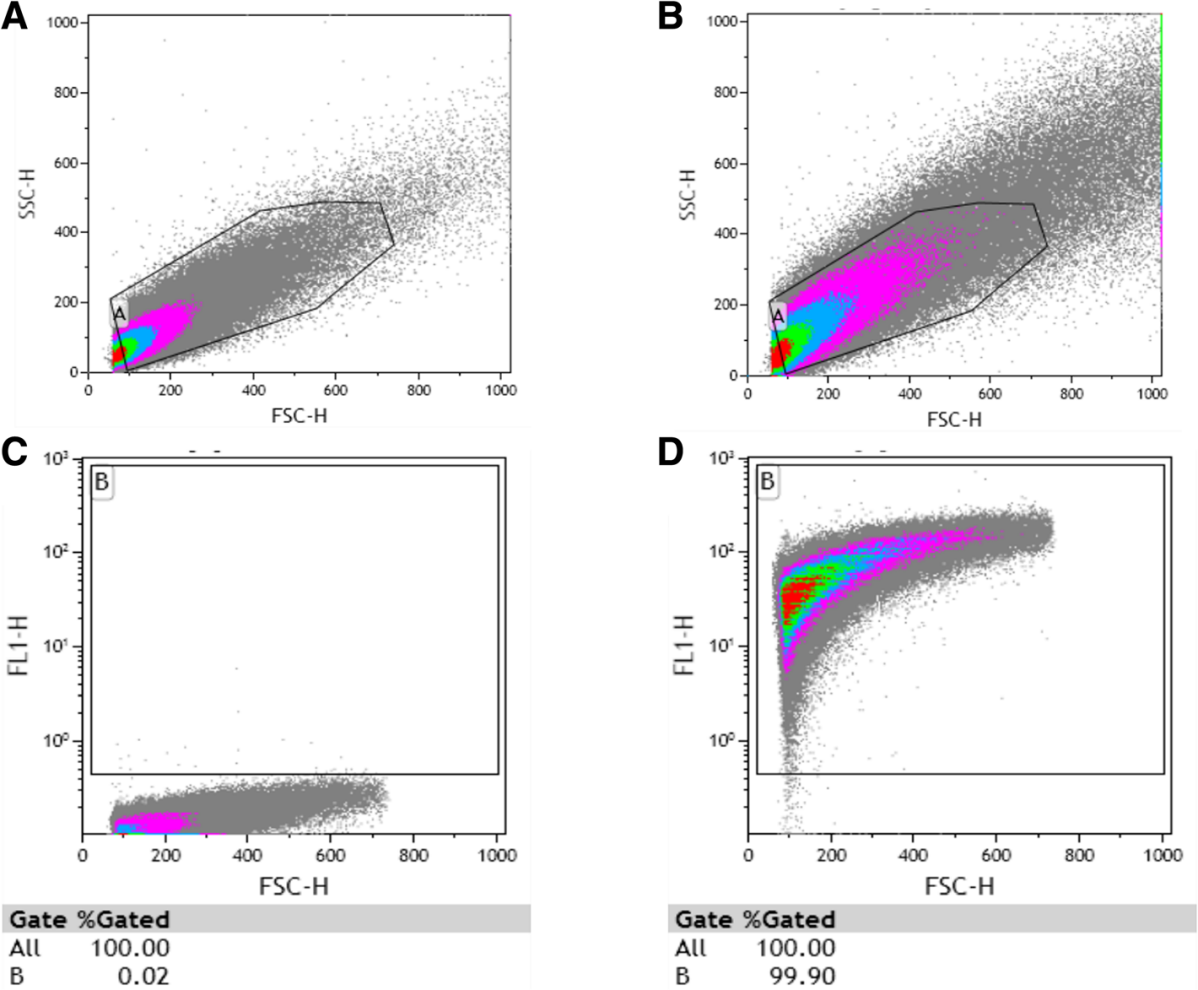
Flow cytometric analysis of CFSE stained *N. gonorrhoeae*. Flow cytometry analysis of non-stained *N. gonorrhoeae* cells forward/side scatter plot (**a**), CFSE-stained *N. gonorrhoeae* forward/side scatter plot (**b**), non-stained *N. gonorrhoeae* forward scatter/FL1 channel plot (**c**) and CFSE-stained *N. gonorrhoeae* forward scatter/FL1 channel plot (**d**). These experiments were performed *n* = 3 times

CFSE staining did not affect viability of *N. gonorrhoeae* and inoculation of GCMBK agar plates with CFSE-stained *N. gonorrhoeae* F62 yielded plentiful pure growth, confirmed by Gram staining (Gram negative diplococcic) and positive oxidase test (data not shown).Forward (FSC-H) and side scatter (SSC-H) dot plot of unstained (Fig. 1a, representative dot plot) and CFSE-stained (Fig. 1b) *N. gonorrhoeae* F62 subjected to flow cytometric analysis is shown in Fig. 1. Viable bacteria are shown in Gate A which confirms that *N. gonorrhoeae* are sufficiently large and complex for them to be detected and quantified using the flow cytometer and that CFSE staining did not impact bacteria size or complexity (Fig. 1b).In Fig. 1c, we show flow cytometric analysis of unstained *N. gonorrhoeae* F62 and Gate B is drawn above the background fluorescence recorded in FL1. Negligible fluorescent events are detected in the unstained bacteria (0.2%; Fig. 1c).When Gate B was applied to the CFSE-stained *N. gonorrhoeae*, 99.9% of the bacteria were fluorescent (Fig. 1d). These results indicate that several thousand *N. gonorrhoeae* (> 30,000) could be quantified using flow cytometric analysis, that CFSE staining does not affect *N. gonorrhoeae* viability, and that CFSE effectively stained the majority of *N. gonorrhoeae*.

### Quantification of adherence of CFSE-labelled *N. gonorrhoeae* to ME-180 cells

**Fig. 2:**
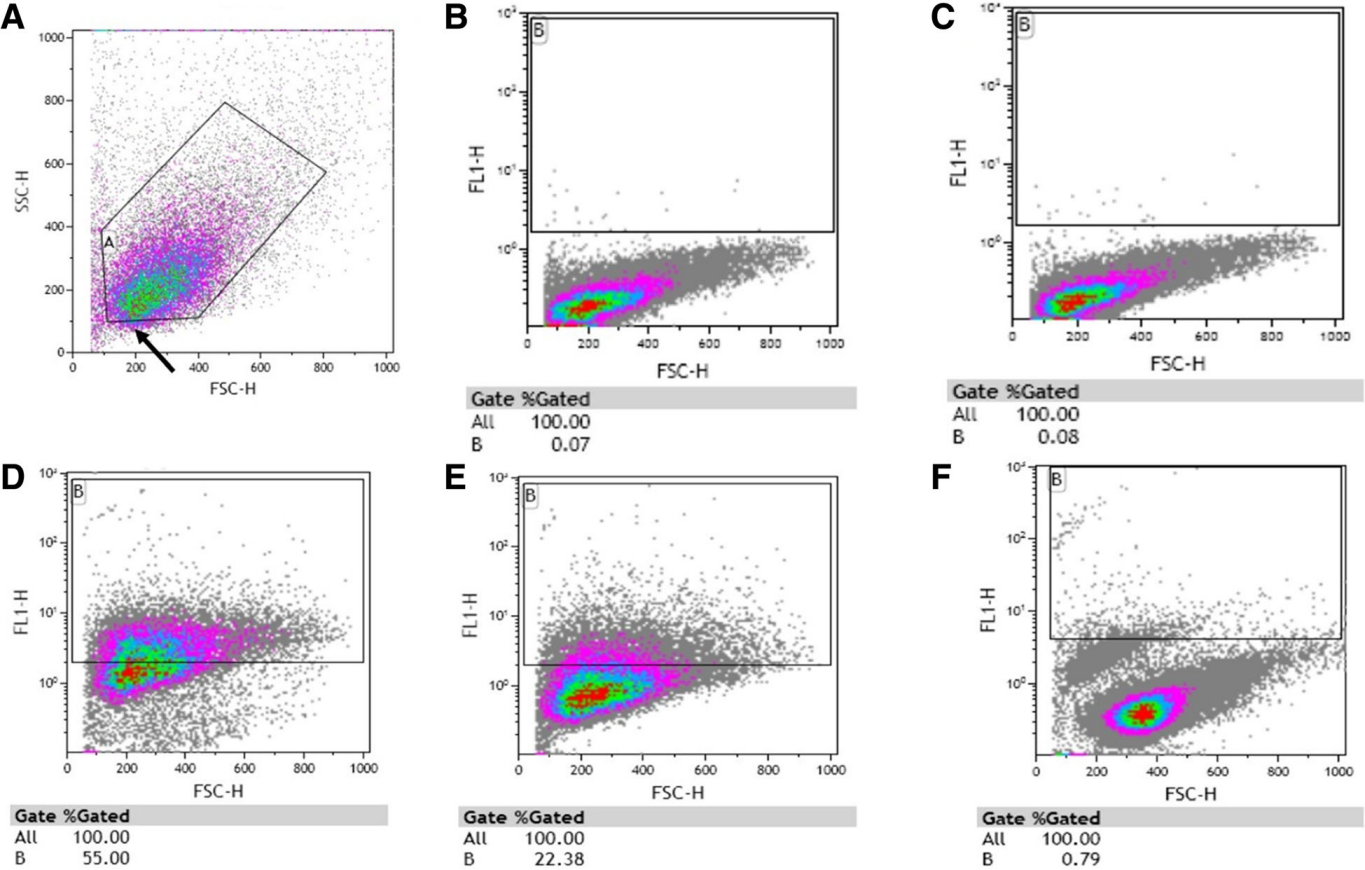
Flow cytometric gating strategy on ME-180 cells. Gating strategy on live ME-180 cells forward/side scatter plot (**a**), ME-180 cells forward scatter/FL1 channel plot (**b**), ME-180 cells infected with non-CFSE stained *N. gonorrhoeae* (MOI 1:100) (**c**), ME-180 cells infected with CFSE-stained *N. gonorrhoeae* (MOI 1:100) without washings with PBS (**d**), ME-180 cells infected with CFSE-stained *N. gonorrhoeae* (MOI 1:100) with washings (*n* = 3) with PBS to remove loosely adhered bacteria (**e**), and ME-180 cells incubated in CFSE-bacterial supernatant forward scatter/FL1 channel plot (**f**)

Figure 2 represents the gating strategies to determine adherence or invasion of CFSE-stained *N. gonorrhoeae* to ME-180 cells. Figure 2a shows the flow cytometric gating strategy for live ME-180 cells (Gate A) whereas Gate B in Fig. 2b-f shows percentage of fluorescent events recorded in FL1-H. As expected, we recorded negligible fluorescence from uninfected ME-180 (0.07%; Fig. 2b) and from ME-180 cells infected with unstained *N. gonorrhoeae* (0.08%; Fig. 2c). As a positive control, ME-180 cells infected with MOI 0.01 CFSE-stained *N. gonorrhoeae* were not washed and 55% fluorescent events were recorded indicating that ME-180 cells were infected with or adhered to by CFSE-stained *N. gonorrhoeae* or that CFSE-stained *N. gonorrhoeae* remained in the culture supernatant. Figure 2e shows a representative plot of ME-180 cells infected with CFSE-stained *N. gonorrhoeae* (MOI 0.01) washed 3 times and 22.38% fluorescent events were recorded (Fig. 2e).These assays were repeated 4 times and an average of 19.2 ± 1.0% fluorescent events were recorded indicating consistent adherence to or infection of ME-180 cells with CFSE-stained *N. gonorrhoeae* (data not shown)*.* To reconfirm purity of the culture, an inoculation of the ME-180 cells infected with CFSE-stained *N. gonorrhoeae* was grown on GCMBK agar after washing and yielded a pure gonococcal growth (data not shown) [11].

Finally, we wished to confirm that any CFSE staining was positively associated with the presence of CFSE-stained bacteria and not from CFSE leaking out from the bacteria and staining the eukaryotic cells. We suspended CFSE-labeled bacteria in cell media for 1 h at 37 °C followed by centrifugation to remove the bacteria and the media was collected. ME-180 cells were then cultured in the media (post-bacterial removal). Flow cytometric analysis of the ME-180 cells showed negligible fluorescence (0.79% fluorescent events in FL-1 channel, Fig. 2f) indicating that the CFSE did not leak out from the bacteria to stain the eukaryotic cells. We can be confident that fluorescent signals detected by flow cytometry indicate the presence of CFSE-bacteria that adhered to or invaded the ME-180 cells.

### Neutralization assay to assess whether serum antibodies blocked *N. gonorrhoeae* adherence of ME-180 cells

**Fig. 3:**
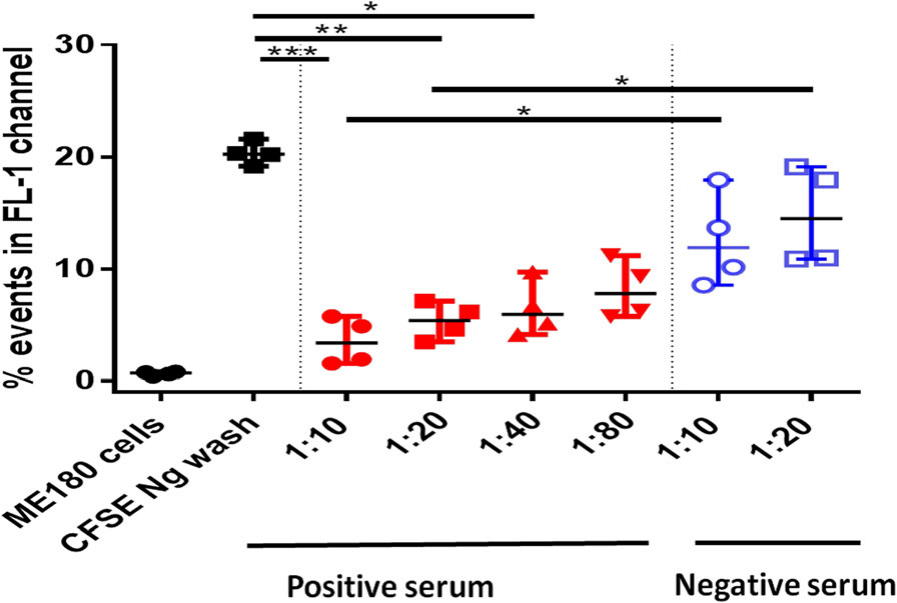
Flow cytometric analysis of neutralization and adherence inhibition assay using ME-180 cells, and ME-180 cells infected with CFSE-stained *N. gonorrhoeae* (Ng) with and without incubation with serum antibodies. CFSE-stained Ng was preincubated with heat inactivated negative (1:10 and 1:20 dilutions) and positive hyperimmune serum (1:10 to 1:80 dilutions). Infected ME-180 cells were washed three times with PBS to remove loosely adherent CFSE-stained Ng

To investigate whether flow cytometric quantification of CFSE-labeled bacteria can be used in conjunction with functional assays, we measured whether antibodies in hyperimmune serum neutralized the adherence of *N. gonorrhoeae* in ME-180 cells. Flow cytometry was performed on ME-180 cells alone or ME-180 cells infected with CFSE-stained bacteria after washing multiple times with saline (Fig. 3). CFSE-stained *N. gonorrhoeae* adhered to or invaded 20.3% (+/− 1.0%) ofME-180cells and pre-incubation of CFSE-stained *N. gonorrhoeae* with 1:10 ratio hyperimmune serum significantly reduced the percentage of events in FL-1 channel (*p* < 0.001), indicating reduced adherence to (or invasion) ME-180 cells. Pre-incubation of CFSE-stained *N. gonorrhoeae* with 1:20(*p* < 0.01) and 1:40 (*p* < 0.05) hyperimmune serum significantly reduced adherence/invasion of ME-180 cells whereas 1:80 hyperimmune serum did not significantly impact adherence/invasion of to bacteria to the eukaryotic cells. Incubation of CFSE-stained *N. gonorrhoeae* with 1:10 (*p* < 0.05) and 1:20 (p < 0.05) control serum prevented significantly less bacterial adherence/invasion of ME-180 cells relative to the corresponding ratio of hyperimmune serum. These data indicate that the hyperimmune serum neutralized *N. gonorrhoeae* adherence/invasion of ME-180 cells and that flow cytometric analysis of bacterial neutralization was highly quantifiable.

### Confirmation of *Neisseria gonorrhoeae* adherence/invasion of ME-180 cells by RT- PCR analysis

**Fig. 4:**
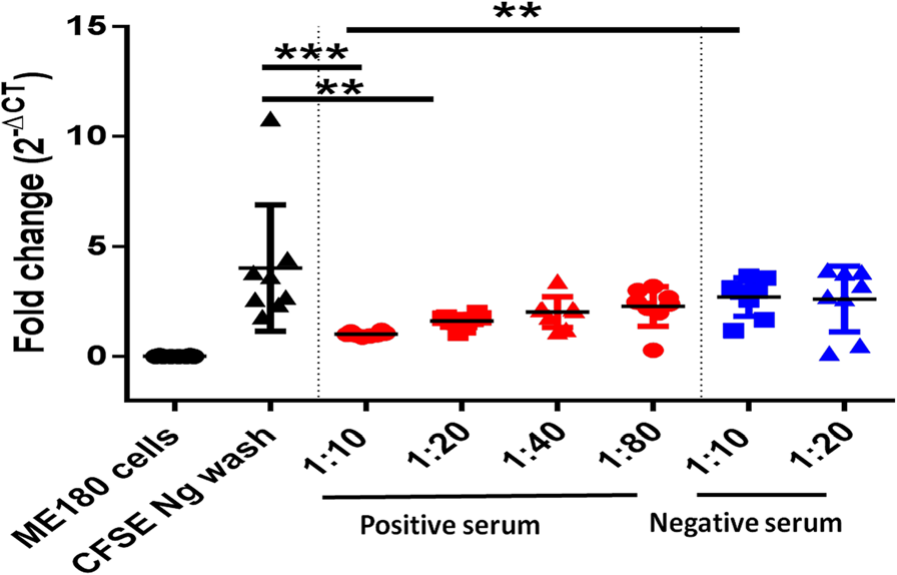
Real Time PCR analysis of neutralization and adherence inhibition assay using ME-180 cells, and ME-180 cells infected with CFSE-stained *N. gonorrhoeae* (Ng) with and without incubation with serum antibodies. CFSE-stained Ng was preincubated with heat inactivated negative (1:10 and 1:20 dilutions) and positive hyperimmune serum (1:10 to 1:80 dilutions). Infected ME-180 cells were washed three times with PBS to remove loosely adherent CFSE-stained Ng

We performed RT-PCR analysis on mock-infected ME-180 cells and ME-180 cells infected with MOI 0.01 CFSE-stained *N. gonorrhoeae* with and without pre-incubation with serum antibodies (Fig. 4). Results indicated that ME-180 cells do not express the bacterial *opa* gene and that CFSE-stained *N. gonorrhoeae* that infected ME-180 cells did express this gene. We inoculated ME-180 cells infected with CFSE-stained *N. gonorrhoeae* on GCMBK agar. As expected, we observed staining with CFSE did not negatively impact *N. gonorrhoeae* growth (data not shown) [11].

Pre-incubation of CFSE-stained *N. gonorrhoeae* with 1:10 (*p* > 0.001) and 1:20 (*p* > 0.01) hyperimmune serum (but not 1:40 and 1:80) significantly reduced the fold-change of *opa* gene expression relative to infected ME-180 cells not pre-treated with hyperimmune serum. The fold-change in *opa* gene expression ME-180 cells infected with CFSE-stained *N. gonorrhoeae* was significantly different (*p* < 0.01) between the bacteria pre-treated with 1:10 ratio hyperimmune serum relative to the control serum. These data indicate that 1:10 ratio of hyperimmune serum blocked the adherence/invasion of CFSE-stained *N. gonorrhoeae* to ME-180 cells. RT-PCR results were in concordance to flow cytometry results and this further establishes that flow cytometry and CFSE staining can be used for *N. gonorrhoeae* adherence/invasion studies.

## Discussion

Detecting *N. gonorrhoeae* adherence of ME-180 cells using flow cytometry has many advantages over traditional culturing and cell-counting methods. This low cost method allows detection of CFSE-stained bacteria inside ME-180 cells without fixation and permeabilization [12].Flow cytometric assay offers a significant increase in quantification capabilities in that it is routinely used to detect 10–100,000 events rather than a mere few hundred which is routinely quantified using conventional techniques. Flow cytometry based assay can detect weak bacterium-cell interactions as compared to conventional solid phase adhesion assays [1]. Tobiason and Seifert (2001) briefly described a CFSE staining-based flow cytometric to measure *N. gonorrhoeae* to human epithelial cell lines (Chang, ME180, HEC-1B and PC-3) [13]. However, we did not come across any study using CFSE staining based flow cytometric to determine *N. gonorrhoeae* adherence to host cells in vitro or in vivo. We made improvements to the method described previously [14] by recording a higher number (30,000) of events compared to this previous study (10,000) and by arresting the CFSE staining of *N. gonorrhoeae* using equal volume of FBS followed by removal of trace amounts of the stain by three washes with PBS. Tobiason and Seifert (2001) did not terminate the fluorescent labelling of *N. gonorrhoeae* and instead simply pelleted the bacteria and washed them once in PBS to remove excess CFDA-SE [13, 14]. This reduced washing and lack of a quenching may have contributed to excessive and non-specific florescence in their study [14]. Further, we used a reduced saponin (0.5%) concentration to lift ME-180 cells associated with fluorescent bacteria from the 24 well plate, compared to previously described method [14]. In our opinion, this contributed to the better collection of ME-180 cells for FACS analysis as it decreased the buoyancy and helped in improved pelleting of the cells during centrifugation. This improvement was reflected by a 3-fold increase in the number of recorded florescent events to 30,000 [14]. We also clarified that ME-180 cells did not acquire CFSE-staining due to the leakage of CFSE from stained bacteria*.* Our results confirm that CFSE-staining of *N. gonorrhoeae* did not trigger any change in colony growth characteristics. The development of an objective and reliable method of quantitating the proportion of epithelial cells with adherent bacteria may be of value in the detailed assessment of the kinetics of distinct myeloid and lymphoid cells responsible for combating *N. gonorrhoeae* infection. For instance, specific cell types infected by *N. gonorrhoeae* and/or recruited to the vagina, fallopian tubes, cervix and draining lymph nodes, could be quantified in early infections (and possibly in late infections if the number of cell divisions has not reduced the amount of CSFE below the level of detection in each daughter cell.) Cell-sorting could then be used to characterize changes in cell function following *N. gonorrhoeae* infection.

## Conclusions

CFSE-staining of *N. gonorrhoeae* is compatible with measuring adherence to ME-180 cells. Flow cytometric analysis and RT-PCR analyses indicated that CFSE- stained *N. gonorrhoeae* adhered to or invaded ME-180 cells and that heat-inactivated rabbit hyperimmune serum inhibited the bacterial adherence/invasion of eukaryotic cells. Together, these findings strongly suggest that CFSE staining can be used in gonococcal adherence/invasion assays.

## Methods

### *N. gonorrhoeae* growth conditions

Frozen stocks (− 80 °C) of *N. gonorrhoeae* F62 isolates were retrieved on Difco™ GC agar medium base (GCMB, BD Biosciences, ON, Canada) supplemented with 1% modified Kellogg’s supplement (GCMBK), at 35 °C in a humid environment with 5–7% CO_2_ [11]. Our F62 strain is part of Dr. Dillon’s personal collection which was originally provided by Dr. Doug Kellogg; information on *N. gonorrhoeae* F62 is provided in [15]. For epithelial cell infections, T1 and T2 colonies were incubated for 12 h and piliated *N. gonorrhoeae* (presence of pili was confirmed with a dissecting microscope) were subcultured on GCMBK [16]. After 10–12 h of incubation growth, piliated *N. gonorrhoeae* were harvested and resuspended in PBS. Cell density was adjusted to an OD_600_ = 0.30 to 0.35 (~ 1 × 10^8^ cfu/ml) for adherence studies [17].

### Cell culture and bacterial propagation conditions

Human cervical ME-180 (ATCC HTB-33) cells were maintained at 37 °C, in a humidified incubator with 5% (*v*/v) CO_2_ in Roswell Park Memorial Institute medium (RPMI) medium 1640 (Gibco #11875–093) containing 5% heat inactivated Fetal Bovine Serum (FBS; Sigma-Aldrich, ON, Canada), 1 mM sodium pyruvate (Gibco # 11360–070) and 1x Anti-Anti (Gibco #15240–062) [10]. Cells were passaged twice weekly in 1:5 ratio in 75 cm^2^ cell culture flasks (Corning Incorporated, Corning, USA).

### CFSE labelling of *N. gonorrhoeae* and flow cytometric analysis

Piliated *N. gonorrhoeae* F62 were stained with CellTrace™ CFSE Cell Proliferation Kit (Molecular Probes, Life Technologies) as described previously [12].To ensure that CFSE staining did not affect viability of *N. gonorrhoeae*, we inoculated GCMBK agar plates with CFSE-stained *N. gonorrhoeae*F62 and plentiful growth was observed. The purity of the CFSE-stained *N. gonorrhoeae* F62 was confirmed by Gram-staining and oxidase testing (data not shown) [11].

### Bacterial adherence assay

Adherence of *N. gonorrhoeae* to ME-180 cells was determined by seeding 2 × 10^5^ ME-180 cells/well in a 24 well tissue culture plate (Costar #3524, Corning Incorporated, Corning, USA) for 18–20 h at 80–90% confluency in antibiotic-free RPMI medium. ME-180 cells were infected with 200 μl (2 × 10^7^ cfu/ml) of CFSE-stained *N. gonorrhoeae* F62 at a multiplicity of infection (MOI) of 1:100 and incubated for 2 h in a humidified chamber, with 5% (*v*/v) CO_2_ at 37 °C [18]. ME-180 cells alone and ME-180 cells infected with non-stained bacteria were used as negative controls. After incubation, infected wells were washed three times with PBS to remove loosely adhered bacteria. As a positive control, select wells containing ME-180 cells infected with CFSE stained bacteria were not washed with PBS. Saponin (500 μl of 0.5% solution in PBS, Sigma-Aldrich, Oakville, ON) was added to each well (37 °C for 15 min) to lift the cells. Wells were washed three times with 500 μl of FACS buffer, the cells were centrifuged at 1000 x g for 10 min then resuspended in 0.4 ml FACS buffer for flow cytometric analysis. All experiments were carried out in duplicate and at least 30,000 events (number of ME-180 cells associated with CFSE stained fluorescent *N. gonorrhoeae*) per well were recorded with a flow cytometer.

### Flow cytometric analysis

Flow cytometric analysis was performed using a BD FACS Calibur™ flow cytometer (BD Biosciences) as detailed in [12] with the exception that uninfected and unstained ME-180 cells were used to establish background fluorescence.

### Animals and generation/validation of hyper immune serum

All animal experimental procedures were performed in accordance with the Procedures for Ethics Review of Animal Use Protocols, and approved by the University Committee on Animal Care and Supply, University of Saskatchewan.

A female New Zealand White rabbit (2–3 kg weight; Charles River Laboratories, Inc.) was used to generate hyperimmune serum specific for *N. gonorrhoeae* F62 as detailed in [12] with the exception that the rabbit was injected with heat-killed (56 °C for 30 min) *N. gonorrhoeae* F62 (1 × 10^8^ cfu/ml suspended in sterile GC broth) and Incomplete Freund’s Adjuvant (Sigma-Aldrich, Oakville, ON) in a 1:1 dilution on day 0, 14, and 28 [17]. Rabbit hyperimmune sera were collected via exsanguination following administration of Euthanyl (Bimeda-MTC Animal Health Inc., Cambridge, ON, Canada) to euthanize the rabbits 42 days after the first vaccination. All blood samples collected in Vacutainers (BD Biosciences-Canada, Mississauga, ON) were centrifuged (2500 rpm for 20 min) and serum was stored at − 20 °C till further use.

The specificity of the hyperimmune serum was determined by Western blot analysis. Briefly, Bicinchoninic acid (BCA) analysis (Pierce BCA Protein Assay Kit, #23225, Thermo Scientific) was performed to quantify protein concentration. Ten μg protein from bacterial cells and ME-180 cells infected for 2 h with *N. gonorrhoeae* F62 was loaded per well and electrophoresed on 10% SDS-PAGE gels. SDS gels were transferred to a nitrocellulose membrane which was hybridized with rabbit anti-*N. gonorrhoeae* hyper immune serum [1:500 in 3% skimmed milk powder (SMP)] at 4 °C overnight. After washing with PBS three times for 10 min intervals, membranes were incubated with goat anti-rabbit IRDye 800CW (LI-COR Biosciences, Lincoln, USA, 1:10,000 in 3% SMP) for 2 h at room temperature. After washing with PBS three times for 10 min intervals, with TBS plus Tween 20. Nitrocellulose membranes were scanned using an Odyssey CLx Infrared Imaging System (LI-COR Biosciences, Lincoln, USA).

The lanes of isolated proteins are as follows: Lane 1- marker proteins, Lane 2- GC broth, Lane 3- ME-180 cells, Lane 4- *N. gonorrhoeae* cells, Lane 5- ME-180 cells infected with *N. gonorrhoeae* (Additional file 1: Figure S1)*.* Antibodies in the hyper immune serum bound to many proteins in lanes 4 and 5 but not in any other lanes indicating specificity for *N. gonorrhoeae* and not ME-180 cells or broth components. The negative control serum failed to bind proteins in any lanes. These data confirmed that the rabbit hyperimmune serum antibodies were specific for *N. gonorrhoeae*.

### Neutralization assay

Ten-fold dilutions of complement-inactivated (56 °C for 30 min) rabbit anti-*N. gonorrhoeae* F62 hyper immune serum and negative rabbit serum (obtained on Day 0 prior to immunization) were prepared in PBS. CFSE-stained *N. gonorrhoeae* F62 (1 × 10^8^ cfu/ml), suspended in PBS, was mixed with 1:1 with 400 μl of each serum dilution followed by incubation at room temperature for 1 h. After incubation, the bacterial suspensions were centrifuged at 2500 x g for 10 min; then, the supernatant was discarded and pellets were resuspended in 200 μl antibiotic-free RPMI at a final concentration of 2 × 10^7^ cfu/ml. Duplicate ME-180 cells (2 × 10^5^ cells/well) were infected serum treated *N. gonorrhoeae* F62 i.e.2 x 10^7^cfu/well. ME-180 cells were either mock-infected (no bacteria; negative control), or infected with CFSE-stained *N. gonorrhoeae* (positive control), CFSE-stained *N. gonorrhoeae* treated with hyper immune serum, CFSE-stained *N. gonorrhoeae* treated with negative control serum. After 2 h, infected ME-180 cells and mock-infected ME-180 cells were collected and prepared for flow cytometric analysis (as indicated above) or Real-time PCR analysis (as detailed below).

### Real time PCR analysis

ME-180 cells +/− CFSE-*N. gonorrhoeae* were subjected to RT-PCR analysis which was performed 5 times with technical duplicates. ME-180 cells+/− CFSE-*N. gonorrhoeae* were collected in 500 μl of 0.5% saponin/well and washed 3 times with PBS, then and pelleted by centrifugation at 1000 x g for 10 min. The cell pellet was ruptured using 250 μl NaOH buffer (25 mM NaOH, 0.2 mM EDTA) and boiling as detailed previously [12]. Real time PCR analysis was performed with primers opa_F (5′-GTTCATCCGCCATATTGTGTTGA-3′) opa_R (5′-AAGGGCGGATTATATCGGGTTCC-3′) targeting *opa gene* using Step One Real-Time PCR System (Applied Biosystems by Life Technologies) after modifying the method described by Donà et al. (2016) [19]. *Opa* gene encodes opacity associated outer membrane proteins which is considered a diagnostic for *N. gonorrhoeae*. Briefly, each 15 μl reaction mixture contained 0.3 M each primer, 2x master mix (Kapa Biosystems, Wilmington, MA), and 2 μl of genomic DNA. The PCR analysis included an initial denaturation step (95 °C for 10 min), followed by 30 cycles of denaturation (95 °C for 15 s), annealing (65 °C for 10 s), and extension (72 °C for 10 s), and produced a specific melt curve for *N. gonorrhoeae* (data not shown).

### Statistical analysis

Statistical analysis and graphing was performed using GraphPad Prism 5 software (GraphPad Software, San Diego, CA). One way ANOVA with Kruskal–Wallis tests were used to compare flow cytometry and qPCR data andmedian values were compared usingDunn’s test. Differences were considered significant if *p* < 0.05.

## Additional file

Additional file 1: **Figure S1.** Western blot analysis with negative (A) and hyperimmune serum (B). 1—ladder; 2 — GC broth; 3 — ME-180 cells; 4 — *N. gonorrhoeae* F62; 5 — ME-180 cells infected with *N. gonorrhoeae* F62. Antibodies to *N. gonorrhoeae* were detected with an IRDye 800CW conjugated goat-anti rabbit antibody. GC broth and ME-180 cells alone were not bound by antibodies for N. gonorrhoeae which indicates that no non-specific binding was observed. Antibodies detected targets in lanes from the bacteria alone and the cells infected with N. gonorrhoeae as anticipated. (TIF 574 kb)

## Abbreviations

CFDA-SE: Carboxyfluoroscein diacetate succinimidyl ester
CFSE: 5′-carboxyfluoroscein succidyl ester
FSC-H: Forward scatter
h: Hour
*N. gonorrhoeae*: *Neisseria gonorrhoeae*
PID: Pelvic inflammatory disease
RT-PCR: Real time-PCR
SSC-H: Side scatter
