# Supplementary figures and images for: Development of flow cytometry based adherence assay for Neisseria gonorrhoeae using 5′-carboxyfluorosceinsuccidyl ester
Source: BMC Microbiol. 2019 Mar 25;19:67. doi: 10.1186/s12866-019-1438-2 (PMC6434840; doi:10.1186/s12866-019-1438-2)

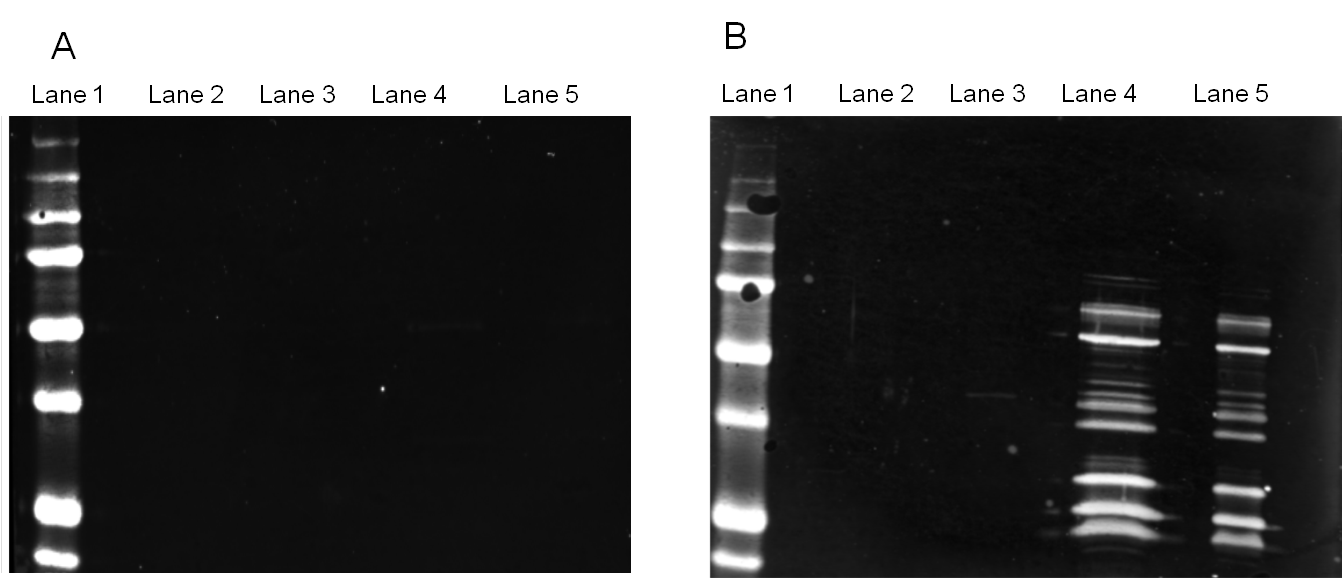

Supplement: Supplementary file 1 — Figure S1. Western blot analysis with negative (A) and hyperimmune serum (B). 1—ladder; 2 — GC broth; 3 — ME-180 cells; 4 — N. gonorrhoeae F62; 5 — ME-180 cells infected with N. gonorrhoeae F62. Antibodies to N. gonorrhoeae were detected with an IRDye 800CW conjugated goat-anti rabbit antibody. GC broth and ME-180 cells alone were not bound by antibodies for N. gonorrhoeae which indicates that no non-specific binding was observed. Antibodies detected targets in lanes from the bacteria alone and the cells infected with N. gonorrhoeae as anticipated. (TIF 574 kb) [file 12866_2019_1438_MOESM1_ESM.tif]
